# Long Non-coding RNAs Responsive to Blast Fungus Infection in Rice

**DOI:** 10.1186/s12284-020-00437-w

**Published:** 2020-11-12

**Authors:** Lan-Lan Wang, Jing-Jing Jin, Li-Hua Li, Shao-Hong Qu

**Affiliations:** 1 Institute of Virology and Biotechnology, Zhejiang Academy of Agricultural Sciences, Hangzhou, 310021 China; 2 China Tobacco Gene Research Center, Zhengzhou Tobacco Research Institute of CNTC, Zhengzhou, 450001 China; 3 School of Plant Protection, Hunan Agriculture University, Changsha, 410128 China

**Keywords:** Rice, Long non-coding RNAs, Rice blast disease, Jasmonate

## Abstract

**Background:**

Long non-coding RNAs (LncRNAs) have emerged as important regulators in many physiological processes in plant. By high-throughput RNA-sequencing, many pathogen-associated LncRNAs were mapped in various plants, and some of them were proved to be involved in plant defense responses. The rice blast disease caused by *Magnaporthe oryzae* (*M. oryzae*) is one of the most destructive diseases in rice. However, *M. oryzae*-induced LncRNAs in rice is yet to be studied.

**Findings:**

We investigated rice LncRNAs that were associated with the rice blast fungus. Totally 83 LncRNAs were up-regulated after blast fungus infection and 78 were down-regulated. Of them, the natural antisense transcripts (NATs) were the most abundant. The expression of some LncRNAs has similar pattern with their host genes or neighboring genes, suggesting a *cis* function of them in regulating gene transcription level. The deferentially expressed (DE) LncRNAs and genes co-expression analysis revealed some LncRNAs were associated with genes known to be involved in pathogen resistance, and these genes were enriched in terpenoid biosynthesis and defense response by Gene Ontology (GO) enrichment analysis. Interestingly, one of up-regulated DE-intronic RNA was derived from a jasmonate (JA) biosynthetic gene, lipoxygenase RLL (LOX-RLL). Levels of JAs were significantly increased after blast fungus infection. Given that JA is known to regulate blast resistance in rice, we suggested that LncRNA may be involved in JA-mediated rice resistance to blast fungus.

**Conclusions:**

This study identified blast fungus-responsive LncRNAs in rice, which provides another layer of candidates that regulate rice and blast fungus interactions.

**Supplementary Information:**

The online version contains supplementary material available at 10.1186/s12284-020-00437-w.

## Findings

Rice is one of the most important foods for humanity and is widely consumed in the world. Rice blast disease caused by the fungus *M. oryzae*, one of the top 10 fungal pathogens (Dean et al. 2012), is the most devastating disease of rice. The global annual crop loss due to blast was estimated at$66 billion and is enough to feed 60 million people (Pennisi 2010).

In order to cope with pathogen infection, rice has evolved a multifaceted, sophisticated defense response to microbial pathogens carrying effectors, as well as pathogen-associated molecular patterns (PAMP) (Liu et al. 2013). The first tier of plant defense is PAMP-triggered immunity (PTI) mediated by pattern recognition receptors and occurs during pathogen attachment and the early phase of host-pathogen interactions. Activation of PTI leads to various defense responses that include the induction of an oxidative burst, activation of mitogen-activated protein kinase (MAPK) cascades, biosynthesis of hormones, accumulation of antimicrobial compounds or enzymes, and callose deposition (involved in the fortification of cell wall); which as a result, inhibits or prevents pathogen proliferation (Mitsuhara et al. 2008; Parker et al. 2009; Shimizu et al. 2010; Bundo and Coca 2015; Yang et al. 2015; Delteil et al. 2016; Urso et al. 2016). The second tier of plant defense is the effectors-triggered immunity (ETI) mediated by plant resistance (R) genes, most of which encode cytoplasmic proteins with nucleotide binding site-leucine-rich repeat (NLR) domains. So far, over 100 major blast R genes have been identified and 30 of them have been molecularly cloned. ETI provides a robust defense response that is often accompanied by a hypersensitive response at the infection site. However, ETI is also race-specific and fragile.

Whole genome RNA sequencing (RNA-Seq), tilling arrays and large-scale cDNA cloning studies have revealed that transcription of eukaryotic genes is highly complex (Forrest and Carninci 2009). The transcriptional landscape in eukaryotes has been extensively studied using RNA-Seq and reveals the RNA molecules are transcribed ranging from protein-coding mRNAs to noncoding transcripts (Chekanova et al. 2007; Berretta and Morillon 2009; Ponting et al. 2009; Sanchez-Leon et al. 2012; Zhu et al. 2014). Noncoding RNAs are classified into two types, containing either short sequences (< 200 nt) or long noncoding RNAs (LncRNAs, longer than 200 nt) (Guttman et al. 2009; Cabili et al. 2011; Wang et al. 2014; Zhou et al. 2014). LncRNA can in turn be classified into long intergenic noncoding RNAs (LincRNA), natural antisense transcripts (NAT) and intronic RNAs (IncRNA) based on genome location (Ponting et al. 2009; Chen 2012; Rinn and Chang 2012; Lina et al. 2013; Dogini et al. 2014). LncRNAs has been revealed to function as key regulators in diverse biological processes, such as flowering time, reproduction, stress responses, organogenesis in roots, and photomorphogenesis in seedlings (Matzke and Mosher 2014; Zhang et al. 2014; Berry and Dean 2015; Li et al. 2016; Wang et al. 2017). Many LncRNAs show significant changes in different organs or during stress, suggesting that they are dynamically regulated and might function in development and stress responses. However, the blast fungus infection-related LncRNAs in rice remains unknown.

**Fig. 1:**
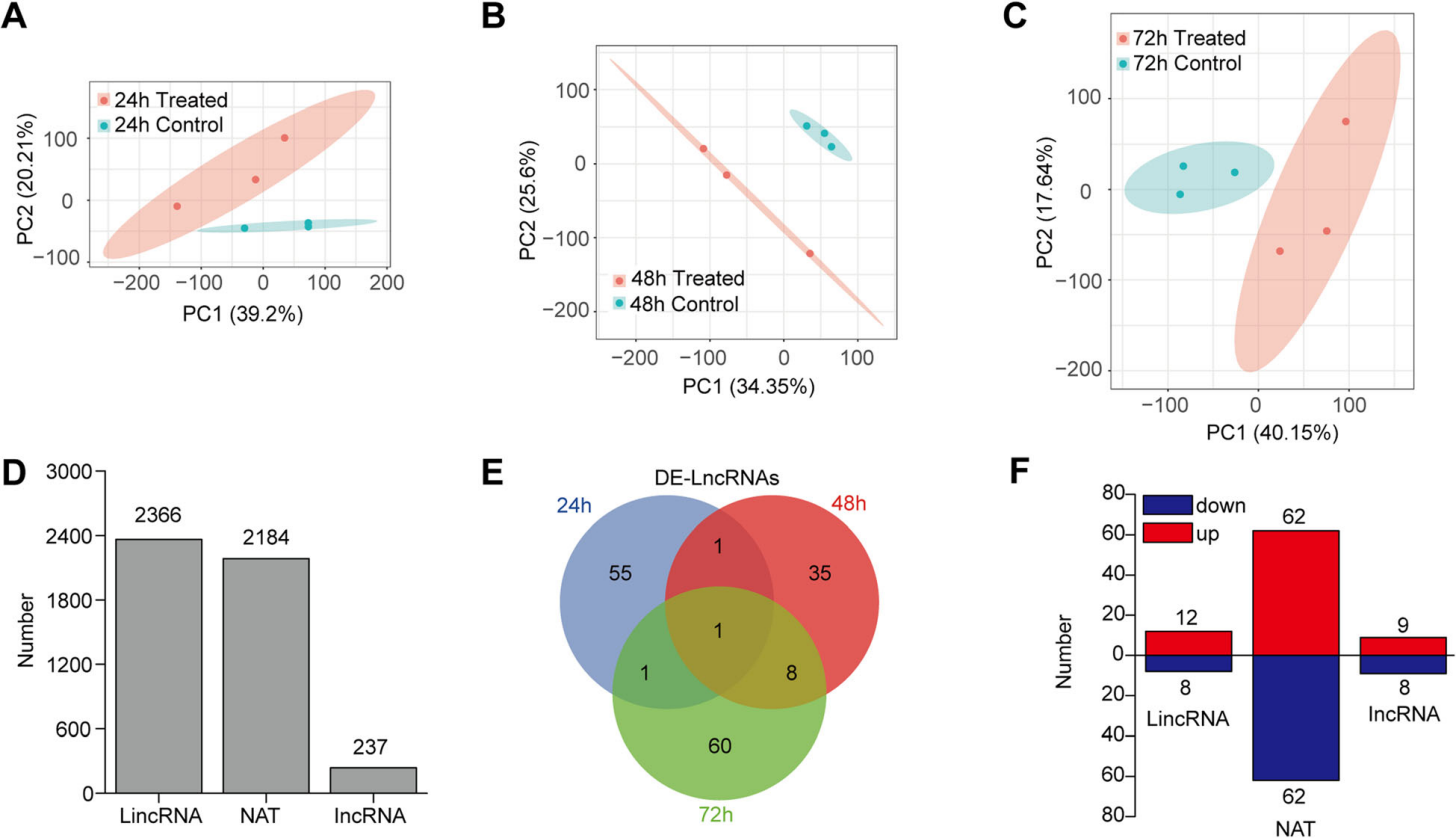
Overview of blast fungus-responsive LncRNAs in rice. **a**-**c** Principal Components analysis (PCA) of mRNA-sequencing data among different treatments. Control, mock treatment. Treated, *M. oryzae* treatment. **d** Total numbers of identified LncRNAs in rice. LincRNA, long intergenic non-coding RNA. NAT, nature antisense transcripts. IncRNA, intronic RNA. **e** A venn diagram showing differentially expressed (DE) LncRNAs 24 h, 48 h and 72 h after *M. oryzae* treatment. Foldchange > 2, *p* value < 0.05. **f** Numbers of up- or down-regulated LncRNAs after blast fungus treatment

**Fig. 2:**
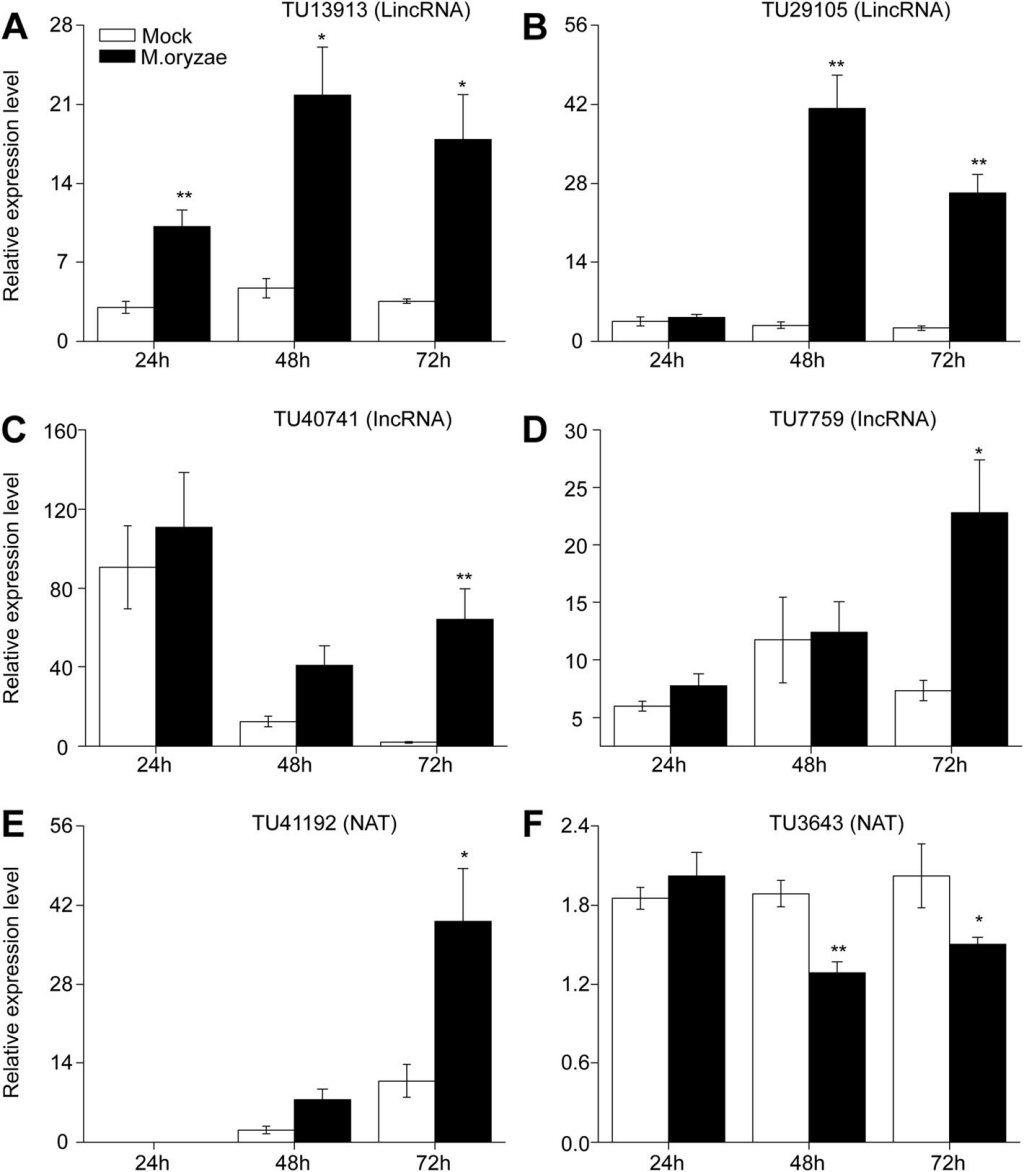
Confirmation of the transcriptional levels of DE-LncRNAs by RT-PCR. Mean transcriptional abundance (±SE, *n* = 5–8) of LincRNAs (**a**, **b**), IncRNAs (**c**, **d**) and NATs (**e**, **f**) in *M. oryzae*-treated samples and control samples. Asterisks indicate significant differences in *M. oryzae*-treated samples compared with control samples (*, *P* < 0.05; **, *P* < 0.01; Student’s t test)

To test if LncRNAs are responsive to blast fungus infection, RNAs of *M. oryzae* treated samples and control were sequenced at three timepoints, each with three replicates. More than 20 million clean reads passed the quality filters (Table S1). These reads were mapped to the rice reference genome, and 95% of the clean reads were aligned for most of the samples. The transcript assembling and expression analysis was then performed. The control and treated group in each timepoint were separated well in principal component analysis (PCA) of the coding RNAs, indicating the significant variation of transcription level after blast fungus treatment (Fig. 1a-c). A total of 4787 transcripts were obtained and defined as LncRNA in rice. Of them, 2366 transcripts were LincRNAs and 2184 transcripts were NATs, while only 237 transcripts were IncRNAs (Fig. 1d). The transcriptional levels of genes and these LncRNAs were compared between blast fungus treated and control samples in each timepoints. A total of 1670 differentially expressed genes (DEGs) and 161 differentially expressed LncRNAs (DE-LncRNAs) were identified (Table S2). One LincNRA was constitutively up-regulated in all of blast fungus treated plants, but other LncRNAs only showed different expression level in a particular timepoint of treatment (Fig. 1e). Among these DE-LncRNAs, about half were up-regulated by blast fungus infection, while the other half was down-regulated (Fig. 1f). To confirm the reliability of the RNA-seq data, six DE-LncRNAs were selected and their transcript levels were confirmed by quantitative RT-PCR (qRT-PCR). The expression of two LincRNAs, TU13913 and TU29105, were significantly higher in blast fungus treated plants compared with control plants (Fig. 2a and b). Two of the IncRNAs, TU40741 and TU7759, were up-regulated at 72 h after blast fungus treatment (Fig. 2c and d). TU41192 was a blast fungus-specific induced NAT, which had a high expression level at 72 h after treatment (Fig. 2e), while another NAT and TU3643 was down-regulated after blast fungus treatment (Fig. 2f). The expression patterns of these LncRNAs done by qRT-PCR were similar with RNA-seq data. A total number of 161 LncRNA transcripts were characterized which showed greater than 2-fold changes (*p* < 0.05) in treated plants compared to control plants.

**Fig. 3:**
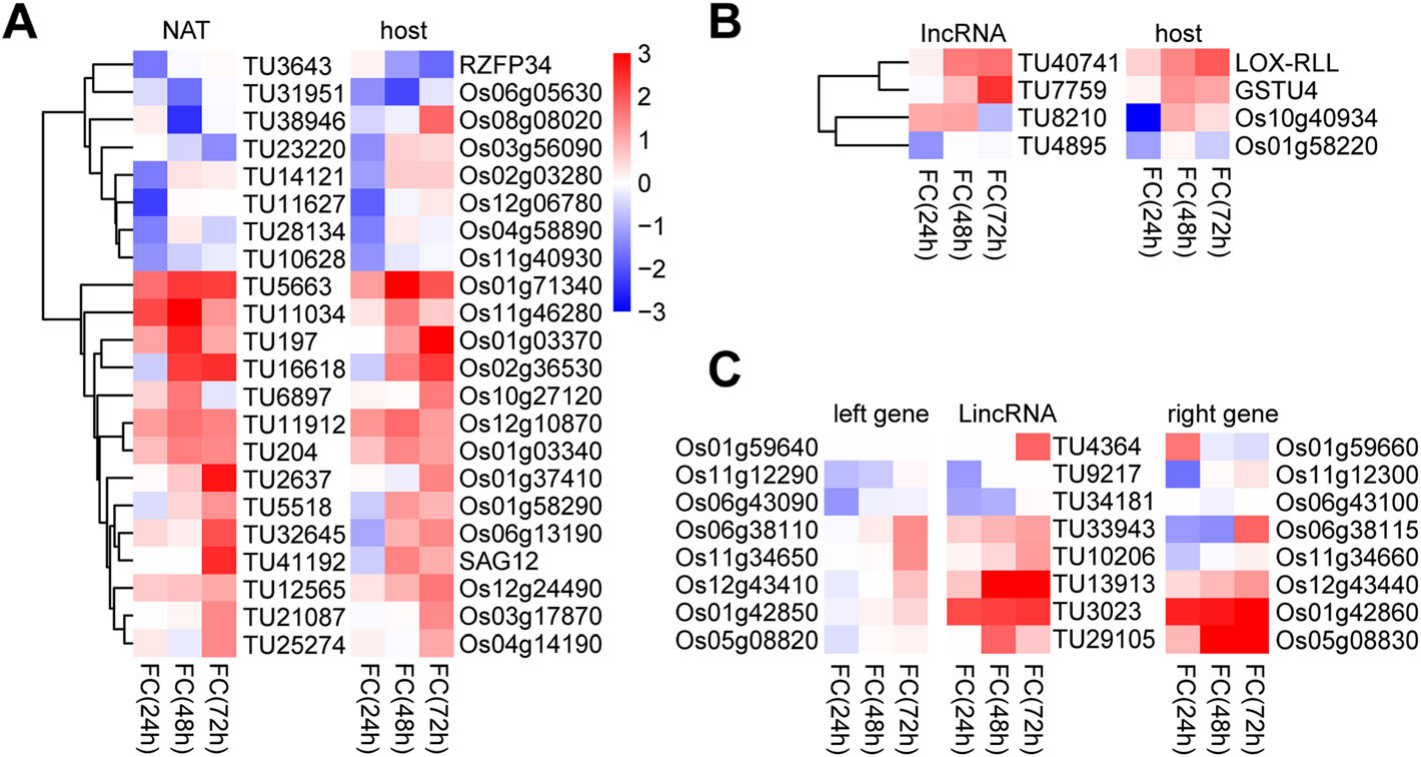
Expression pattern of blast fungus-responsive LncRNAs and their host or neighboring genes. Heatmap representing the transcript abundance fold change (FC) of NAT (**a**), IncRNA (**b**) and their host gene in *M. oryzae*-treated samples compared with control samples (**c**). Heatmap representing the transcript abundance fold change of LincRNA and its neighboring gene in *M. oryzae*-treated samples compared with control samples. U, up-regulated; D, down-regulated

The functions of these DE-LncRNAs in plant and blast fungus interaction were predicted. First, some LncRNAs act as cis element to regulate transcription machinery and chromatin modification on the promoter region the expression of nearby genes (Liu et al. 2015). For instance, the LncRNA COOLAIR and COLDAIR is derived from antisense and intron region of FLOWERING LOCUS C (FLC) gene in Arabidopsis (Heo and Sung 2011). Both LncRNAs could directly interact with Polycomb-Responsive Complex 2 to repress the transcription levels of FLC. On the other hand, LncRNA could also directly bind to mediator complex to activate the expression of neighboring gene in mammal (Lai et al. 2013). The LincRNA ELENA1 in Arabidopsis could also interact with Mediator subunit 19a to activate PR1 expression upon pathogen attack, although it acts as a *trans* element (Seo et al. 2017). To predict if the blast fungus-induced LncRNAs have cis-functions, the expression of DE-LncRNA and their nearby gene were compared. The host genes of NATs and IncRNAs and the neighboring genes of LincRNAs were firstly identified. The transcription levels of these genes with at least 2-fold change between treated and control samples were screened. Totally 34 DE-LncRNA-gene pairs were identified (Fig. 3a, b and c). Of them, most of LncRNAs and their associated genes showed the same expression trend. For example, the NAT TU3643 was down-regulated by blast fungus treatment, and its host gene, RZFP34 (RING zinc-finger protein 34), was also down-regulated (Figs. 2f and 3a). RZFP34 was known to regulate stomata opening in rice (Hsu et al. 2014). Many plant pathogens including the rice blast fungus gain entry to their host via stomata, suggesting TU3643-RZFP34 may be involved in rice and blast fungus interaction. The expression of NAT TU41192 and its host gene SAG12 (senescence-associated gene) were both up-regulated by blast fungus treatment (Figs. 2e and 3a). SAG12 negatively regulate stress-induced cell death which may also play a role in blast fungus resistance (Singh et al. 2016). A gutathione S-transferase gene, GSTU4, plays a role in plant tolerance to oxidative stresses (Sharma et al. 2014). The expression of an IncRNA TU7759 and its host gene GSTU4 have similar pattern in different treatment (Fig. 3b). Interestingly, a jasmonate biosynthetic gene, lipoxygenase RLL (LOX-RLL) and its intronic RNA TU40741 were also up-regulated by blast fungus treatment.

**Fig. 4:**
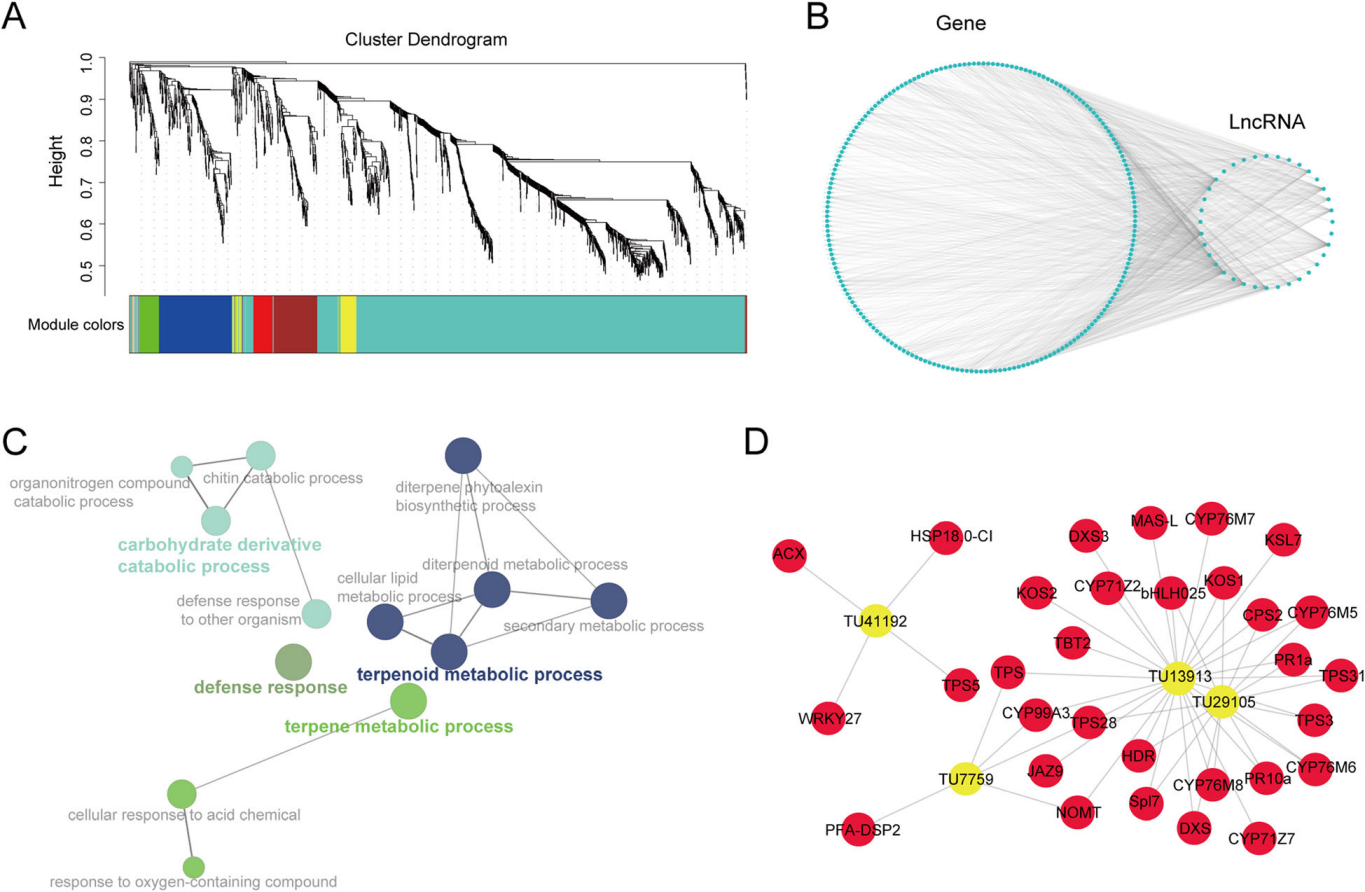
Co-expression analysis blast fungus-responsive genes and LncRNAs and GO enrichments of genes associated with LncRNAs. **a** Weighted correlation network analysis (WGCNA) of deferentially expressed (DE) LncRNAs and mRNAs. For DE-genes, we used the cutoff: foldchange > 2, Adjust *p* value < 0.05. Colors represent modules generated by WGCNA. **b** Network of module turquoise on the basis of WGCNA analysis. We used the cutoff: weight > 0.4. **c** Gene Ontology (GO) enrichment analysis of genes selected in (**b**). The size of character represents the significance of the GO; Color of the bubble represents GO group. **d** Network plot of correlation between four DE-LncRNAs and known pathogen resistance-related genes. The information of these blast pathogen resistance-related genes were listed in Table S3

To explore the connection between DE-LncRNAs and DEGs, weighted correlation network analysis (WGCNA) was performed (Langfelder and Horvath 2008). The co-expressed transcripts were clustered into 7 modules (Fig. 4a). Each module was indicated by different color, and the module turquoise is the most abundant with 1270 module members. We screened the high correlated connection in module turquoise by using weight higher than 0.4. A total of 203 coding genes and 35 LncRNAs were selected, and the co-expression network was constructed (Fig. 4b). To detect which genes were associated with these LncRNAs, the gene ontology (GO) enrichment analysis was performed. Genes involved in defense response and terpenoid metabolic processes were significantly enriched (Fig. 4c). Terpenoid, especially diterpenes, are well-known to be involved in rice resistance to blast fungus (Chen et al. 2018). The network between four LncRNAs (in Fig. 2 and module turquoise) and the known pathogen resistance-related genes were picked out (Fig. 4d). These LncRNAs were co-expressed with diterpene biosynthetic genes, jasmonate signaling pathway genes, pathogenesis-related genes, and transcription factors (Table S3). These results suggested that DE-LncRNAs may function in rice-blast fungus interaction.

**Fig. 5:**
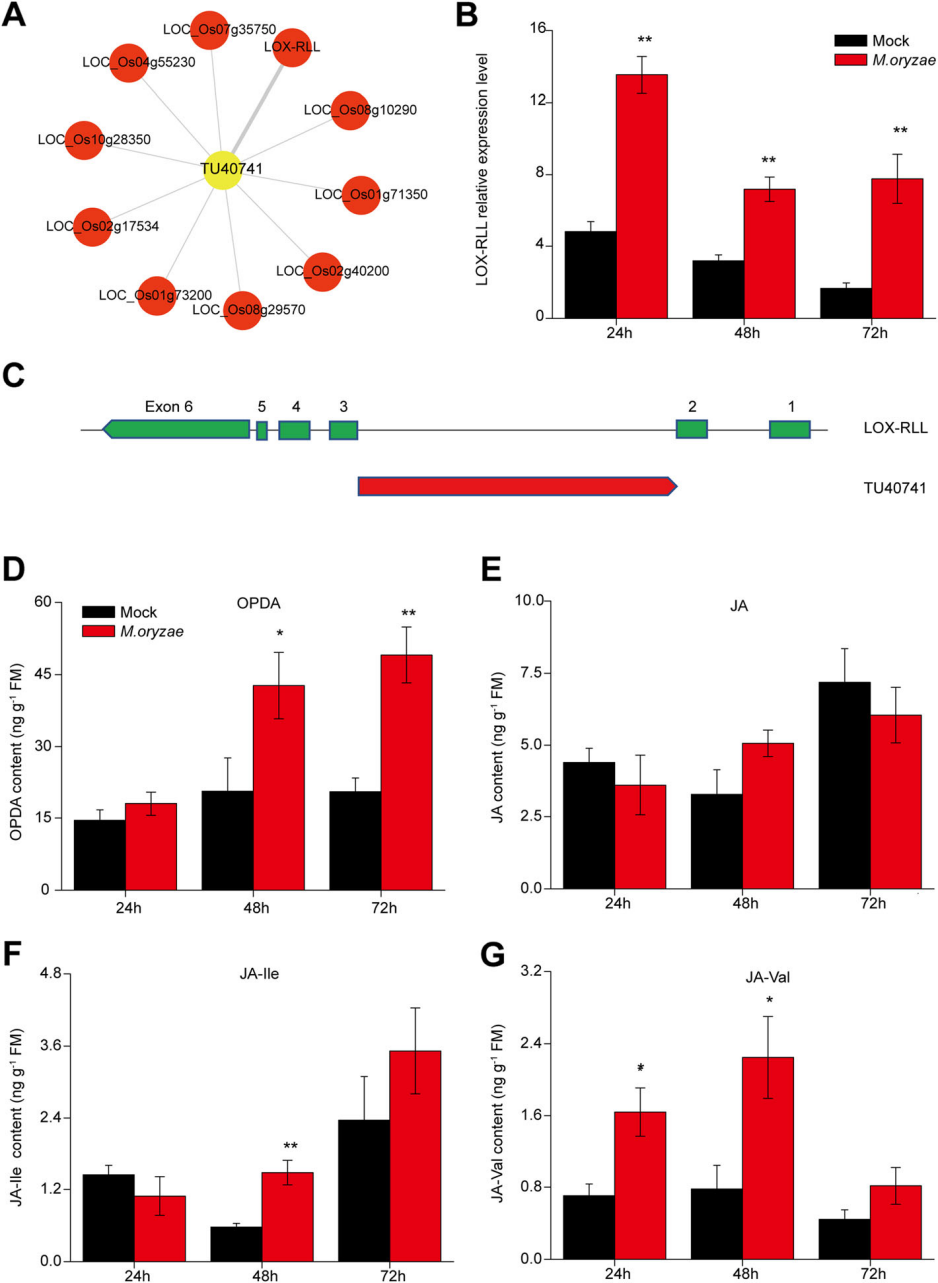
An intronic RNA is derived from jasmonate biosynthetic gene. **a** Network plot of correlation between a DE-IncRNA and co-expressed genes based on WGCNA analysis. LOX-RLL, herbivore induced lipoxygenase. **b** Mean transcriptional abundance (±SE, *n* = 5–8) of LOX-RLL in *M. oryzae*-treated samples and control samples. Asterisks indicate significant differences in *M. oryzae*-treated samples compared with control samples (**, *P* < 0.01; Student’s t test). **c** Schematic diagram of LOX-RLL and its intron-derived transcripts. The six exons of LOX-RLL were highlighted by green. The intronic RNA was derived between exon 2 and exon 3, which has a opposite direction compared with LOX-RLL transcripts. Mean levels (±SE, *n* = 5–8) of OPDA (**d**), JA (**e**), JA-Ile (**f**) and JA-Val (**g**) in *M. oryzae*-treated samples and control samples. Asterisks indicate significant differences in *M. oryzae*-treated samples compared with control samples (**, *P* < 0.01; Student’s t test)

TU40741 is an intronic RNA which is derived from a jasmonate (JA) biosynthetic gene, LOX-RLL. Through the network analysis from module yellow, TU40741 was also highly co-expressed with LOX-RLL gene (Fig. 5a). LOX-RLL together with AOS (allene oxide synthase) and AOC (allene oxide cyclase) catalyze linolenic acid to 12-oxo-phytodienoic acid (OPDA), the precursor of jasmonic acid. LOX-RLL transcripts were increased in blast fungus treated plants compared with control plants by qRT-PCR analysis (Fig. 5b). Sequence analysis revealed that TU40741 is derived from the second intron of LOX-RLL and has an opposite transcriptional direction with LOX-RLL gene (Fig. 5c). JA signaling pathway is known to regulate rice resistance to blast fungus (Rakwal and Komatsu 2000; Jwa et al. 2001; Riemann et al. 2013; Shimizu et al. 2013; Yang et al. 2013; Urso et al. 2016). The products levels of LOX-RLL were measured after blast fungus infection. The levels of OPDA were significantly increased 48 h and 72 h after blast fungus treatment (Fig. 5d). However, no significant difference was observed for jasmonic acid levels (Fig. 5e), consistent with previous study (Riemann et al. 2013). The downstream bioactive forms of jasmonate, jasmonoyl-isoleucine (JA-Ile) and jasmonoyl-valine (JA-Val) levels were both enhanced in treated plants (Fig. 5f and g). The terpenoids were known to function as antimicrobial phytoalexins in rice (Schmelz et al. 2015). Some of monoterpene and diterpenoid are involved in rice resistance to blast fungus (Chen et al. 2018). Interestingly, the biosynthesis of these antifungal compounds is regulated by JA signaling (Riemann et al. 2013; Chen et al. 2018). LOX-RLL has been long time identified as *M. oryzae-*induced gene in rice (Peng et al. 1994). LOX-RLL was also known as herbivore-induced LOX (HI-LOX). Silencing of HI-LOX in rice decreased herbivore-induced JA levels and made plants more susceptible to chewing herbivores (Zhou et al. 2009), suggesting the role of LOX-RLL in biotic stress induced JA signaling. These data suggested that LOX-RLL-mediated JA biosynthesis may play a role in blast fungus resistance. Some intronic RNAs are characterized to regulate the expression of their host genes (Heo and Sung 2011). Thus, we inferred TU40741 may regulate the expression of LOX-RLL, which in turn mediate JA signaling pathway. Emerged evidences have showed that LncRNAs were associated with pathogen-induced JA biosynthesis. Two LncRNA, GhlncNAT-ANX2- and GhlncNAT-RLP7, regulate the expression of LOX1 and LOX2 gene in cotton after fungal *Verticillium dahliae* attack (Zhang et al. 2017). In rice, the lncRNA ALEX1 activates JA pathway and plant resistance to bacterial blast (Yu et al. 2020). Further study is required to elucidate the role of LOX-RLL and TU40741 in rice defense against blast fungus.

In conclusion, we identified blast fungus-induced LncRNAs in rice by high-throughput RNA-sequencing. By co-expression analysis, some LncRNAs were predicted to be highly correlated with pathogen resistance. Strikingly, an intronic RNA was characterized to connect with blast-fungus induced JA signaling pathway. Our results provide novel candidates for the regulation study of rice-blast fungus interaction.

## Supplementary Information

**Additional file 1:** **Table S1.** Summary of LncRNA-sequencing used in this study.

**Additional file 2:** **Table S2.** LncRNAs responsive to *M. oryzae* infection.

**Additional file 3:** **Table S3.** The information of blast pathogen resistance-related genes and associated LncRNAs in Fig. 4.

**Additional file 4:** **Table S4.** Primers used in qRT-PCR.

**Additional file 5.** Materials and Methods.

## Data Availability

The raw RNA-sequencing data reported in this paper have been deposited in the Genome Sequence Archive in BIG Data Center, Beijing Institute of Genomics (BIG), Chinese Academy of Sciences, under accession numbers CRA003133.

## Abbreviations

AOS: Allene oxide synthase
AOC: Allene oxide cyclase
DE: Deferentially expressed
ETI: Effectors-triggered immunity
FLC: FLOWERING LOCUS C
GSTU4: Gutathione S-transferase gene
GO: Gene ontology
HI-LOX: Herbivore-induced lipoxygenase
IncRNAs: Intronic RNAs
JA: Jasmonate
JA-Ile: Jasmonoyl-isoleucine
JA-Val: Jasmonoyl-valine
JAR1: Jasmonic acid resistance 1
LDMAR: LONG-DAY-SPECIFIC MALE-FERTILITY-ASSOCIATED RNA
LincRNAs: Long intergenic noncoding RNAs
LncRNAs: Long non-coding RNAs
LOX-RLL: Lipoxygenase RLL
NATs: Natural antisense transcripts
NLR: Nucleotide binding site-leucine-rich repeat
OPDA: 12-oxo-phytodienoic acid
PAMP: Pathogen-associated molecular patterns
PTI: PAMP-triggered immunity
PCA: Principal component analysis
R: Resistance
SAG12: Senescence-associated gene
WGCNA: Weighted correlation network analysis

## References

[CR1] Berretta J, Morillon A (2009). Pervasive transcription constitutes a new level of eukaryotic genome regulation. EMBO Rep.

[CR2] Berry S, Dean C (2015). Environmental perception and epigenetic memory: mechanistic insight through FLC. Plant J.

[CR3] Bundo M, Coca M (2015). Enhancing blast disease resistance by overexpression of the calcium-dependent protein kinase OsCPK4 in rice. Plant Biotechnol J.

[CR4] Cabili MN, Trapnell C, Goff L, Koziol M, Tazon-Vega B, Regev A, Rinn JL (2011). Integrative annotation of human large intergenic noncoding RNAs reveals global properties and specific subclasses. Genes Dev.

[CR5] Chekanova JA, Gregory BD, Reverdatto SV, Chen H, Kumar R, Hooker T, Yazaki J, Li P, Skiba N, Peng Q (2007). Genome-wide high-resolution mapping of exosome substrates reveals hidden features in the Arabidopsis transcriptome. Cell.

[CR6] Chen X (2012). Small RNAs in development-insights from plants. Curr Opin Genet Dev.

[CR7] Chen X, Chen H, Yuan JS, Kllner TG, Chen F (2018). The Rice terpene synthase gene OsTPS19 functions as an (S)-limonene synthase in planta and its overexpression leads to enhanced resistance to the blast fungus Magnaporthe oryzae. Plant Biotechnol J.

[CR8] Dean R, Kan JALV, Pretorius ZA, Hammond-Kosack KE, Pietro AD, Spanu PD, Rudd JJ, Dickman M, Kahmann R, Ellis J (2012). The top 10 fungal pathogens in molecular plant pathology. Mol Plant Pathol.

[CR9] Delteil A, Gobbato E, Cayrol B, Estevan J, Michel-Romiti C, Dievart A, Kroj T, Morel JB (2016). Several wall-associated kinases participate positively and negatively in basal defense against rice blast fungus. BMC Plant Biol.

[CR10] Dogini DB, Pascoal VDAB, Avansini SH, Vieira AS, Lopes-Cendes I (2014). The new world of RNAs. Genet Mol Biol.

[CR11] Forrest ARR, Carninci P (2009). Whole genome transcriptome analysis. RNA Biol.

[CR12] Guttman M, Amit I, Garber M, French C, Lin MF, Feldser D, Huarte M, Zuk O, Carey BW, Cassady JP (2009). Chromatin signature reveals over a thousand highly conserved large non-coding RNAs in mammals. Nature.

[CR13] Heo JB, Sung S (2011). Vernalization-mediated epigenetic silencing by a long intronic noncoding RNA. Science.

[CR14] Hsu KH, Liu CC, Wu SJ, Kuo YY (2014). Expression of a gene encoding a rice RING zinc-finger protein, OsRZFP34, enhances stomata opening. Plant Mol Biol.

[CR15] Jwa NS, Agrawal GK, Rakwal R, Park CH, Agrawal VP (2001). Molecular cloning and characterization of a novel jasmonate-inducuble pathogenesis-realted class 10 protein gene, JIOsPR10, from rice (Oryza sativa L.) seedling leaves. Biochem Biophys Res Commun.

[CR16] Lai F, Orom UA, Cesaroni M, Beringer M, Taatjes DJ, Blobel GA, Shiekhattar R (2013). Activating RNAs associate with mediator to enhance chromatin architecture and transcription. Nature.

[CR17] Langfelder P, Horvath S (2008). WGCNA: an R package for weighted correlation network analysis. BMC Bioinformatics.

[CR18] Li S, Yamada M, Han X, Ohler U, Benfey PN (2016). High-resolution expression map of the Arabidopsis root reveals alternative splicing and lincRNA regulation. Dev Cell.

[CR19] Lina M, Vladimir BB, Zhang Z (2013). On the classification of long non-coding RNAs. RNA Biol.

[CR20] Liu J, Wang H, Chua NH (2015). Long noncoding RNA transcriptome of plants. Plant Biotechnol J.

[CR21] Liu W, Liu J, Ning Y, Ding B, Wang X, Wang Z, Wang GL (2013). Recent progress in understanding PAMP- and effector-triggered immunity against the rice blast fungus Magnaporthe oryzae. Mol Plant.

[CR22] Matzke MA, Mosher RA (2014). RNA-directed DNA methylation: an epigenetic pathway of increasing complexity. Nat Rev Genet.

[CR23] Mitsuhara I, Iwai T, Seo S, Yanagawa Y, Kawahigasi H, Hirose S, Ohkawa Y, Ohashi Y (2008). Characteristic expression of twelve rice PR1 family genes in response to pathogen infection, wounding, and defense-related signal compounds (121/180). Mol Genet Genomics.

[CR24] Parker D, Beckmann M, Zubair H, Enot DP, Caracuel-Rios Z, Overy DP, Snowdon S, Talbot NJ, Draper J (2009). Metabolomic analysis reveals a common pattern of metabolic re-programming during invasion of three host plant species by Magnaporthe grisea. Plant J.

[CR25] Peng Y, Shirano Y, Hiroyuki O, Takashi H, Kunisuke T, Daisuke S (1994). A novel lipoxygenase from rice. J Biol Chem.

[CR26] Pennisi E (2010). Armed and dangerous. Science.

[CR27] Ponting CP, Oliver PL, Reik W (2009). Evolution and functions of long noncoding RNAs. Cell.

[CR28] Rakwal R, Komatsu S (2000). Role of jasmonate in the rice (Oryza sativa L.) self-defense mechanism using proteome analysis. Electrophoresis.

[CR29] Riemann M, Haga K, Shimizu T, Okada K, Ando S, Mochizuki S, Nishizawa Y, Yamanouchi U, Nick P, Yano M (2013). Identification of rice allene oxide cyclase mutants and the function of jasmonate for defence against Magnaporthe oryzae. Plant J.

[CR30] Rinn JL, Chang HY (2012). Genome regulation by long noncoding RNAs. Annu Rev Biochem.

[CR31] Sanchez-Leon N, Arteaga-Vazquez M, Alvarez-Mejia C, Mendiola-Soto J, Duran-Figueroa N, Rodriguez-Leal D, Rodriguez-Arevalo I (2012). Transcriptional analysis of the Arabidopsis ovule by massively parallel signature sequenicng. J Exp Bot.

[CR32] Schmelz EA, Huffaker A, Sims JW, Christensen SA, Lu X, Okada K, Peters RJ (2015). Biosynthesis, elicitation and roles of monocot terpenoid phytoalexins. Plant J.

[CR33] Seo JS, Sun HX, Park BS, Huang CH, Yeh SD, Jung C, Chua NH (2017). ELF18-INDUCED LONG-NONCODING RNA associates with mediator to enhance expression of innate immune response genes in Arabidopsis. Plant Cell.

[CR34] Sharma R, Sahoo A, Devendran R, Jain M (2014). Over-expression of a rice tau class glutathione S-transferase gene improves tolerance to salinity and oxidative stresses in Arabidopsis. PLoS One.

[CR35] Shimizu T, Miyamoto K, Miyamoto K, Minami E, Nishizawa Y, Iino M, Nojiri H, Yamane H, Okada K (2013). OsJAR1 contributes mainly to biosynthesis of the stress-induced Jasmonoyl-isoleucine involved in defense responses in rice. J Agric Chem Soc Japan.

[CR36] Shimizu T, Nakano T, Takamizawa D, Desaki Y, Ishii-Minami N, Nishizawa Y, Minami E, Okada K, Yamane H, Kaku H, Shibuya N (2010). Two LysM receptor molecules, CEBiP and OsCERK1, cooperatively regulate chitin elicitor signaling in rice. Plant J.

[CR37] Singh S, Singh A, Nandi AK (2016). The rice OsSAG12-2 gene codes for a functional protease that negatively regulates stress-induced cell death. J Biosci.

[CR38] Urso S, Desiderio F, Biselli C, Bagnaresi P, Crispino L, Piffanelli P, Abbruscato P, Assenza F, Guarnieri G, Cattivelli L, Vale G (2016). Genetic analysis of durable resistance to Magnaporthe oryzae in the rice accession Gigante Vercelli identified two blast resistance loci. Mol Gen Genomics.

[CR39] Wang D, Qu Z, Yang L, Zhang Q, Liu Z-H (2017). Transposable elements (TEs) contribute to stress-related long intergenic noncoding RNAs in plants. Plant J.

[CR40] Wang H, Chung PJ, Liu J, Jang IC, Chua NH (2014). Genome-wide identification of long noncoding natural antisense transcripts and their responses to light in Arabidopsis. Genome Res.

[CR41] Yang DL, Yang Y, He Z (2013). Roles of plant hormones and their interplay in rice immunity. Mol Plant.

[CR42] Yang Z, Ma H, Hong H, Yao W, Xie W, Xiao J, Li X, Wang S (2015). Transcriptome-based analysis of mitogen-activated protein kinase cascades in the rice response to Xanthomonas oryzae infection. Rice.

[CR43] Yu Y, Zhou Y, Feng Y, He H, Lian J, Yang Y, Lei M, Zhang Y, Chen Y (2020). Transcriptional landscape of pathogen-responsive lncRNAs in rice unveils the role of ALEX1 in jasmonate pathway and disease resistance. Plant Biotechnol J.

[CR44] Zhang L, Wang M, Li N, Wang H, Qiu P, Pei L, Xu Z, Wang T, Gao E, Liu J (2017). Long noncoding RNAs involve in resistance to Verticillium dahliae, a fungal disease in cotton. Plant Biotechnol J.

[CR45] Zhang YC, Liao JY, Li ZY, Yu Y, Zhang JP, Li QF, Qu LH, Shu WS, Chen YQ (2014). Genome-wide screening and functional analysis identify a large number of long noncoding RNAs involved in the sexual reproduction of rice. Genome Biol.

[CR46] Zhou G, Qi J, Ren N, Cheng J, Erb M, Mao B, Lou Y (2009). Silencing OsHI‐LOX makes rice more susceptible to chewing herbivores, but enhances resistance to a phloem feeder. Plant J.

[CR47] Zhou ZY, Li AM, Adeola AC, Liu YH, Irwin DM, Xie HB, Zhang YP (2014). Genome-wide identification of long intergenic noncoding RNA genes and their potential association with domestication in pigs. Genome Biol Evol.

[CR48] Zhu QH, Stephen S, Taylor J, Helliwell CA, Wang MB (2014). Long noncoding RNAs responsive to Fusarium oxysporum infection in Arabidopsis thaliana. New Phytol.

