# Supplementary material for: Long Non-coding RNAs Responsive to Blast Fungus Infection in Rice
Source: Rice (N Y). 2020 Nov 12;13:77. doi: 10.1186/s12284-020-00437-w (PMC7661613; doi:10.1186/s12284-020-00437-w)
Supplement: Supplementary file 1 — Additional file 1: Table S1. Summary of LncRNA-sequencing used in this study. [file 12284_2020_437_MOESM1_ESM.docx]

Table S1 Summary of LncRNA-sequencing used in this study.

| sample | num of reads | map rate | pair map rate |
| --- | --- | --- | --- |
| 24h-treated-1 | 220449228 | 98.76% | 96.14% |
| 24h-treated-2 | 248531341 | 98.42% | 95.37% |
| 24h-treated-3 | 165412261 | 98.39% | 95.14% |
| 24h-control-1 | 287388631 | 98.94% | 96.58% |
| 24h-control-2 | 232635088 | 98.87% | 96.47% |
| 24h-control-3 | 210630496 | 98.95% | 96.57% |
| 48h-treated-1 | 192429383 | 97.57% | 94.20% |
| 48h-treated-2 | 224322740 | 98.04% | 94.99% |
| 48h-treated-3 | 152808176 | 97.88% | 93.93% |
| 48h-control-1 | 188829023 | 98.40% | 95.56% |
| 48h-control-2 | 221173701 | 98.57% | 95.82% |
| 48h-control-3 | 193195101 | 98.39% | 95.54% |
| 72h-treated-1 | 174323858 | 97.72% | 94.63% |
| 72h-treated-2 | 199961690 | 98.08% | 95.23% |
| 72h-treated-3 | 183565623 | 97.94% | 94.75% |
| 72h-control-1 | 161431237 | 98.39% | 95.62% |
| 72h-control-2 | 205681888 | 96.28% | 91.98% |
| 72h-control-3 | 241368090 | 98.07% | 94.77% |
