# Supplementary material for: Long Non-coding RNAs Responsive to Blast Fungus Infection in Rice
Source: Rice (N Y). 2020 Nov 12;13:77. doi: 10.1186/s12284-020-00437-w (PMC7661613; doi:10.1186/s12284-020-00437-w)
Supplement: Supplementary file 3 — Additional file 3: Table S3. The information of blast pathogen resistance-related genes and associated LncRNAs in Fig. 4. [file 12284_2020_437_MOESM3_ESM.docx]

Table S3 The information of blast pathogen resistance-related genes and associated LncRNAs in Fig. 4

| Name | Gene ID | Associated LncRNAs |
| --- | --- | --- |
| DXS | LOC_Os01g01710 | TU13913, TU29105 |
| bHLH025 | LOC_Os01g09990 | TU13913, TU29105 |
| WRKY27 | LOC_Os01g40430 | TU41192 |
| TPS3 | LOC_Os02g02930 | TU13913, TU29105 |
| TPS5 | LOC_Os02g36020 | TU41192 |
| CPS2 | LOC_Os02g36210 | TU13913, TU29105 |
| HDR | LOC_Os02g39160 | TU13913, TU29105 |
| PFA-DSP2 | LOC_Os02g53160 | TU7759 |
| HSP18.0-CI | LOC_Os03g16030 | TU41192 |
| TPS | LOC_Os03g24760 | TU7759, TU13913 |
| CYP99A3 | LOC_Os04g09920 | TU7759, TU13913 |
| MAS-L | LOC_Os04g33240 | TU13913 |
| Spl7 | LOC_Os05g45410 | TU7759, TU13913 |
| ACX | LOC_Os06g23780 | TU41192 |
| KOS2 | LOC_Os06g37224 | TU13913 |
| KOS1 | LOC_Os06g37300 | TU7759, TU13913 |
| PR1a | LOC_Os07g03710 | TU7759, TU13913 |
| DXS3 | LOC_Os07g09190 | TU13913 |
| CYP71Z2 | LOC_Os07g11739 | TU13913 |
| TPS28 | LOC_Os07g11790 | TU7759, TU29105 |
| TPS31 | LOC_Os08g07100 | TU13913, TU29105 |
| NOMT | LOC_Os12g13800 | TU7759, TU13913 |
| PR10a | LOC_Os12g36880 | TU13913, TU29105 |
| CYP76M5 | LOC_Os02g36030 | TU13913, TU29105 |
| CYP76M6 | LOC_Os02g36280 | TU13913, TU29105 |
| CYP76M7 | LOC_Os02g36110 | TU13913 |
| CYP76M8 | LOC_Os02g36070 | TU13913, TU29105 |
| TPS3 | LOC_Os02g02930 | TU13913, TU29105 |
| TPS31 | LOC_Os08g07100 | TU13913, TU29105 |
| CYP71Z7 | LOC_Os02g36190 | TU13913 |
| TBT2 | LOC_Os11g42370 | TU13913 |
