# Supplementary material for: Long Non-coding RNAs Responsive to Blast Fungus Infection in Rice
Source: Rice (N Y). 2020 Nov 12;13:77. doi: 10.1186/s12284-020-00437-w (PMC7661613; doi:10.1186/s12284-020-00437-w)
Supplement: Supplementary file 4 — Additional file 4: Table S4. Primers used in qRT-PCR. [file 12284_2020_437_MOESM4_ESM.docx]

Table S4 Primers used in qRT-PCR

| Gen ID | F | R |
| --- | --- | --- |
| TU40741 | GGGGCGACTTTAAGTTTGGT | TCTATCCGAAGGGAAACACG |
| TU3643 | TTCAGATGCGAGTGATTTGC | GTGGCGAAGTTTTGATGTGA |
| TU41192 | GGTTGAACTCGTGGATGAGG | GAGTTCGGCGACAAGGAC |
| TU13913 | CCCAACTTCGTCTCTTCTCG | GGCCTTGAAATATGGCTGAC |
| TU7759 | CTCTGTTCCGCTCTCTCACC | AGGAGCTCGCTCTTGTTGAC |
| TU29105 | TCGCGTTAGATGATGGACAG | TGAGGCAGAGGCAAAACTCT |
| LOX-RLL | AAAAGGACAAGTTCGCATGG | TGGAAGTCGAGCATGAACAG |
