# Supplementary material for: Long Non-coding RNAs Responsive to Blast Fungus Infection in Rice
Source: Rice (N Y). 2020 Nov 12;13:77. doi: 10.1186/s12284-020-00437-w (PMC7661613; doi:10.1186/s12284-020-00437-w)
Supplement: Supplementary file 5 — Additional file 5. Materials and Methods. [file 12284_2020_437_MOESM5_ESM.docx]

**Long non-coding RNAs responsive to blast fungus infection in rice**

Lan-Lan Wang ^1, *^, Jing-Jing Jin ^2^, Li-Hua Li^1,3^, Shao-Hong Qu ^1*^

^1^ Institute of Virology and Biotechnology, Zhejiang Academy of Agricultural Sciences, 310021 Hangzhou, China

^2^ China Tobacco Gene Research Center, Zhengzhou Tobacco Research Institute of CNTC, 450001 Zhengzhou, China

3 School of plant protection, Hunan Agriculture University, 410128, Changsha, China

^*^ To whom correspondence should be addressed.

**Materials and Methods**

**Plant material and fungal inoculation**

Rice (*Oryza sativa* ssp *japonica* cv Nipponbare) were surface-sterilized and transferred to MS medium. After germination, rice seedlings were transplanted into vermiculite-filled pots and kept in a growth chamber at 26 °C/24 °C under a 14 h light/10 h dark cycle with 85% humidity. The *M. oryzae* strains (TMC-1) was cultured on complete medium (Zhou et al. 2016) at 25°C for 5-6 days from storage at -20°C. Then 5×10^5^ spores/mL with 0.02% Tween-20 was used as final concentration for inoculation.

When two fully expanded leaves appeared, whole plants were inoculated using spraying infection method with the spores of TMC, and the inoculated plants were kept in a dark chamber at 85% humidity and 24 °C. After 34 hpi, the plants were maintained in the growth chamber at 26 °C/24 °C in a 14 h light/12 h dark cycle with 85% humidity. Leaves were collected at 24, 48, and 72 hours post inoculation. Rice plants were treated with 0.02% Tween-20 and collected at the same time points for use as mock treatments. Six to eight plants for each treatment were used. All samples were immediately frozen in liquid nitrogen and kept at −80 °C until further use.

**LncRNA sequencing**

LncRNA sequencing was performed by Novogene Company (Beijing, China). In brief, the total RNA without rRNA was used to construct the strand-specific RNA libraries with an insert size of 250-300 bp. The paired-end 150 bp sequencing was then performed on an Illumina Hiseq platform. Three replicates for each treatment were performed.

**Transcriptome assembly and annotations**

For the RNA-seq data, all sequenced reads from each experiment were trimmed using trim galore program (https://www.bioinformatics.babraham.ac.uk/projects/trim_galore/ ) with a quality score 30, then, clean data were aligned to the rice reference genome (http://rice.plantbiology.msu.edu/) using the read aligner HIAST2(Kim et al. 2015). The transcriptome of each experiment was assembled separately using stringtie (Pertea et al. 2015), all gtf result files were merged into one with stringtie --merge. Then, we compared the assembled transcript isoforms with the rice genome reference annotation information, which represents all protein coding gene models identified by rice genome project (http://rice.plantbiology.msu.edu/). Transcripts with a length shorter than 200bp and an open reading frame (ORF) length longer than 120 aa were discarded (ORF Finder, links), in order to remove transcripts that may encode short protein, swiss-prot database were searched using blastx program with the parameter -e 1.0e-4 -S 1. The CPC (Li et al. 2014)programs were used to calculate the coding potential of the remaining transcripts. Only transcripts with both CPC scores less than 0 were used for the subsequent analysis. The remaining transcripts located in intergenic regions were identified as lincRNA candidates. If the transcripts were transcribed from the antisense strands of known genes, they will be considered as NATs candidates. For the transcripts located in intron region of known genes, they were identified as incRNA candidates. The expression level of all locus identified including lncRNAs and protein-coding genes was using TPM by stringtie(Pertea et al. 2015).

**Quantitative Real-time PCR (qRT-PCR)**

Total RNA was extracted from the leaf blades of rice after treatment using MiniBEST Plant RNA Extraction Kit (Takara, Janpan) including DNase I treatment according to the manufacturer’s instructions. RNA samples with 260/280nm ratio between 2.0-2.2, and RNA integrity number greater than 8.0, were used for the analysis. Reverse transcription was performed using 2μg of each total RNA and oligo (dt) primers by the PrimerScript RT Master Mix (Takara, Janpan). Six to eight independent biological samples were collected and analyzed. The relative quantity of lncRNAs and genes were performed using qRT-PCR. It was performed using SYBR Premix Ex TaqTM II Kit (Takara, shiga, Janpan) on the Bio-Rad CFX96 real-time system with gene-specific primers. Primer sequences used are listed in Supplemental Table S4.

**Differential expression analysis**

The log-transformed TPM (Trans Per Million) value was used for transcriptional level. To get the differentially expressed genes and lncRNAs, all of the transcripts from pairwise samples were screened using the adjust p value less than 0.05 and fold change higher than 1.

**Co-expression analysis**

The differentially expressed genes and lncRNAs were used for co-expression analysis.

Subsequent weighted gene co-expression network analysis was conducted using the R package WGCNA (Langfelder et al. 2008). The network of each module was visualized using Cytoscape (Ono et al. 2014).

**PCA analysis**

The log-transformed TPM (Trans Per Million) values of all coding genes were used for principle component analysis (PCA). PCA was performed using R package factoextra.

**Heatmap analysis**

The value of fold change in each timepoint was used for heatmap analysis. Heatmap was performed by R package pheatmap.

**GO enrichment analysis**

Gene ontology (GO) analysis was performed by ClueGO (Bindea et al. 2009).

Pathway with p value less than 0.05 was showed.

**JA analysis**

Approximately 100mg material was used for JA analysis. Samples were ground and extracted with 800 μL of ethyl acetate containing the internal standards (10 ng of D6-JA and 10 ng of D6-JA-Ile). Extracts were evaporated and dissolved with 300 μL of 70 % methanol. All samples were analyzed by UHPLC-HESI-MS/MS as previously described (Li et al. 2017).

**References**

Bindea G, Mlecnik B, Hackl H, Charoentong P, Galon J (2009). ClueGO. Bioinformatics **25**: 1091-1093

Kim D, Langmead B, Salzberg SL (2015). HISAT: a fast spliced aligner with low memory requirements. Nature Methods **12**: 357-360

Langfelder P, Horvath S (2008). WGCNA: an R package for weighted correlation network analysis. BMC Bioinformatics **9**: 1-13

Li A, Zhang J, Zhou Z (2014). PLEK: a tool for predicting long non-coding RNAs and messenger RNAs based on an improved k-mer scheme. BMC Bioinformatics **15**: 1471-2105

Li R, Wang M, Wang Y, Schuman MC, Baldwin IT (2017). Flower-specific jasmonate signaling regulates constitutive floral defenses in wild tobacco. Proceedings of the National Academy of Ences of the United States of America **114**: E7205-E7214

Ono K, Demchak B, Ideker T (2014). Cytoscape tools for the web age: D3.js and Cytoscape.js exporters. F1000research

Pertea M, Pertea GM, Antonescu CM, Chang TC, Mendell JT, Salzberg SL (2015). StringTie enables improved reconstruction of a transcriptome from RNA-seq reads. Nature Biotechnology **33**: 290-295

Zhou XG, Yu P, Dong C, Yao CX, Zhao ZW (2016). Proteomic analysis of mycelial proteins from Magnaporthe oryzae under nitrogen starvation. Genetics & Molecular Research Gmr **15**
